# Notes and Comments

**Published:** 1923-08

**Authors:** 


### NOTES AND COMMENTS.

### THE COTTAGE HOSPITAL.

WE have dealt at length in our first leading article with Sir Napier Burnett's annual report on the provincial hospitals; but he has certain things to say regarding the cottage hospitals to which it is desirable that separate attention should be drawn. His facts and figures suggest that these institutions are on their trial. They have done, and are doing, admirable work, but with a reasonable amount of co-ordination they would much more completely fulfil their destiny. That 1,839 out of 4,572 beds in these 314 hospitals should be unoccupied on the average all the year round is distinctly disastrous. At the present time a use can be found for every hospital bed in the country, and it is discouraging indeed to learn that, in consequence of the low occupation, the daily cost of a patient in many small hospitals is higher than in some of the Medical School hospitals with all their elaborate equipment. This is a grotesque position which must not be allowed to continue. Sir Napier Burnett suggests that some of the empty beds might be used for maternity cases from outlying districts, and for patients of the great hospitals in early stages of convalescence. The Medical School hospitals are overpressed, and most of them have waiting lists which might sensibly be relieved if cottage hospitals would place some empty beds at their disposal. Much of the usefulness of a cottage hospital depends upon its medical staff, and in towns of substantial size it not unfrequently happens that it has at its command such first-rate surgical skill that it is not under the necessity of sending difficult cases to the nearest great hospital. And it must not be forgotten that some first-rate general hospitals began life as cottage hospitals.

### THE PLUS AND THE MINUS OF SUNSHINE.

THE sun, we know, is the friend of man, though, for a masculine luminary, his ways are exceedingly feminine. For the greater part of the present year he has been more than usually coy—the heavens have been his pouting place. Quite suddenly, and without warning, in the early part of July, he determined to give a taste of his quality to the luckless people who had been speaking disrespectfully of him. He beamed upon them with the utmost geniality, with consequences not dissimilar from those suggested by the smile on the face of the historic tiger. Yet in "larning" us to be toads he rather overshot the mark and produced certain results which had not been in the programme. He made the fortune of every ice manufacturer in a week, and secured first-class dividends for linen-drapers who had been obsessed with gloomy thoughts of putting up the shutters. Ice could not be made fast enough, and the shops were speedily emptied of their "frocks" and diaphanous blouses. It is true that some of those who could not obtain ice or afford organdie died of the heat, and that a good many others were made very ill by being sent to the tropics when they had firmly believed (consult the school books *passim*) that they lived in a temperate climate. We now know better what is meant by a temperate zone—it is one in which one extreme of temperature succeeds another, and in which the transition is heralded by a discharge of heavenly artillery that awakens every man under fifty to a belief that he is still in the trenches. But at least the old French gibe about our English summer—three fine days and a thunderstorm—has at last been disproved. Three thunderstorms and a week of eighty or ninety in the shade is the new reading, and for some time to come no well-educated Englishman will complain of grey days and a low thermometer. Like the reformed malefactor who knew that honesty was the best policy because he had tried both, he is now satisfied that unseasonable coolness is vastly preferable to "seasonable" sunshine.

### THE MINISTER'S SURVEY.

HOWEVER novel to the general public may have been many of the things which Mr. Neville Chamberlain had to say upon the vote for the Ministry of Health, most of his statement was necessarily already familiar to our readers. It was, on the whole, a satisfactory statement. It showed that there is a progressive improvement in the public health, with a lower death-rate and especially a lessened mortality among very young children. Indeed, as the Minister put it in an arresting phrase, "the baby born to-day can reasonably expect to live twelve years longer than its grandfather." For all this we have chiefly to thank preventive work; yet we are only at the beginning of what preventive medicine is likely presently to accomplish. Last year sickness, among the insured population alone, caused the loss of twenty million weeks of work, and if to this figure we add the incapacity of the uninsured, we reach a total which tells us that we have a long row to hoe before any Minister of Health can be really jubilant. There is, however, solid reason for congratulation in the fact that, during a series of exceptionally difficult years, we have done something far better than mark time. Mr. Chamberlain emphasised the point, to which we have more than once called attention, that hospital extension is overdue, and that until further provision has been made the voluntary system "cannot be said finally to have made that system good."

### HOSPITALS AND INFIRMARIES.

MR. NEVILLE CHAMBERLAIN, in the course of his speech, gave his countenance to the proposal that the Poor Law Infirmaries, with their many vacant beds, should come to the rescue of the voluntary hospitals, just as Sir Napier Burnett has suggested that the cottage hospitals should help them. The Labour Party, by the voice of Mr. Arthur Henderson, goes much farther and demands that the sick should be taken out of the Poor Law and the Infirmaries taken over by the State. Mr. Henderson would like the Government to "act at once," and when one London hospital has 300 patients waiting for fifteen beds, impatience is excusable. But the subject is by no means so simple as it seems. The Infirmaries are supported out of the rates; if the Government took them over they would be supported out of the taxes. This looks uncommonly like the thin end of the wedge for the creation of State hospitals. That because a patient goes to an Infirmary he should be classed as a pauper is entirely undesirable, and to that extent we are in agreement with Mr. Henderson. But why should not the Infirmaries be taken over for the use of panel patients? Sooner or later we shall have to get rid of the stigma of pauperism in sickness, and this suggestion is at least worth discussion. The one thing upon which no finger must be laid is the voluntary hospital system.

### A CENSORSHIP OF MODERN REMEDIES.

If we were to accept as true a mere fraction of the claims of the manufacturing chemist, the road to complete health would be as short as the road to the chemist's shop. But as even manufacturing chemists are only human, and apt to claim greater merits for their products than the patient or his physician can discover, it would be a good plan if new remedies had to run the gauntlet of a Board of Examiners not composed solely of manufacturing chemists. This idea has recently taken practical form in Germany—the land of the synthetic, proprietary drug, the birthplace of salvarsan, and of many less potent, but equally secret, remedies. The German Medical Society has co-operated in this matter with the insurance societies, chemists' associations and other bodies, and a scheme has been evolved according to which manufacturers will be encouraged to submit their new products to chemical, pharmacological and clinical tests. The clinical tests are to be carried out in hospitals, where the conditions are, of course, more favourable than in general practice. The movement is still in its infancy, and its viability will doubtless depend on the impartiality with which its sponsors' views are translated into practice. If it succeeds in giving prominence to remedies which are not only intrinsically beneficial, but are sold at a reasonable price, it will be doing yeoman service to the community.

### CONTROLLING INFECTIOUS DISEASE IN CANADA.

The annual report for 1922 of the activities of the nurses connected with the Victorian Order of Nurses for Canada, issued by the Chief Superintendent, Mrs. Hanington, is a record of progress in all directions. The increase of 4,532 over the number of patients cared for in the previous year, and of 88,317 more visits paid speaks for itself as to the energy and diligence of the nurses in the various branches of educational and preventive work in which they are engaged. A new departure has been made in the direction of dealing with communicable diseases, which include 1,387 cases. This has been most successful. It is now the duty of the nurse in such cases to see herself that proper quarantine is maintained, that the patient receives adequate attention, and that the other members of the family and community generally are protected from infection. In one instance, a nurse took four small-pox cases out of a rural school, obtained medical aid and supervised the cases in their homes, with the result that no other cases developed. An illustrated account of the rise and development of the Victorian Order of Nurses for Canada appeared in our issue for October 29, 1921.

### LADY ASTOR'S BILL.

Lady Astor's Intoxicating Liquor Bill, which, as it now stands, prevents the sale of alcoholic drinks to young persons under eighteen years of age, has passed the House of Commons and has obtained its second reading in the Lords. A reasonable exception is made for boys and girls who are having a meal—they may drink with it beer, stout, cider or perry. We must get rid of the unhappy habit of youths standing about in bars, and growing familiar with the public-house atmosphere. The Bill is a useful step forward, but we are very slow in arriving at the reformed public-house. Every year, however, sees a good many licensed houses absorbed by the various Trusts which aim at conducting refreshment houses rather than drinking shops. Meanwhile we are sorry that cider should have been placed in the category of alcohol. It is, we believe, possible, by strict attention to business, to "get forrarder" on cider, but the average cyclist or pedestrian rarely ever comes into contact with the forward variety.

### LEGACIES TO HOSPITALS.

That, when a legacy is bequeathed to a hospital, the State should take toll of it precisely as it would if it enabled a private individual to keep a motor-car or refrain from work for the rest of his life, not only looks, but is, exceedingly mean. Viscount Ednam, in the House of Commons and a representative deputation outside it, have asked the Government to forgo an impost which produces no more than £150,000 or £200,000 a year. The Financial Secretary to the Treasury was, however, adamant on the subject. He argued that if the concession were made, the Government would be pressed to extend it to other charities. No doubt they would; but, from the charitable point of view, hospitals stand alone, and it would be perfectly easy for the Government to say so. We recognise that the time is unpropitious for asking for remissions of taxation, but the sum which the Treasury would lose by ceasing to levy legacy duty upon bequests to hospitals would be almost negligible, while the hospitals would benefit substantially. A little more imagination in official quarters would be a blessing indeed, and it might have been supposed that even a Treasury whose business it is to be hard-hearted would have been prepared a little further to help the Eldest Daughter of the Nation—the Hospital.

### RADIUM AND LIFE CHANGES.

A remarkably interesting paper from Dr. Herman Lawrence appears in a recent number of the *Medical Journal of Australia*, in which he recounts experiments showing the effect of radium emanations upon a silk-worm colony, and applies the results to human treatment. The experiments have continued over ten years, and they certainly provide justifica- for using radium in the treatment of superficial malignant growths. Of more than secondary interest, however, are the curious results produced upon the insects and their eggs. Radium emanation is found readily to destroy fertilised eggs if the exposure lasts long enough, and is, in this respect, much more potent than the commoner X-rays.

On the other hand, if a batch of unfertilised eggs is briefly exposed to radium, Dr. Lawrence claims that occasionally it is possible to hatch one of them out, a living though not very perfect male insect being produced. The exact nature of the radio-stimulus which can thus produce fertilisation is, of course, quite unknown, but it seems that on occasion it succeeds. We note another important point in these records. One of the criticisms of radium treatment is that it sometimes stimulates rather than kills a growth. This has often been denied by those who should know, and Dr. Lawrence finds that when silkworm larvæ, seeds, vegetables, grasses, etc., are exposed to radium emanations, he has never obtained any evidence whatever that their rate and power of growth have been stimulated. Always the living thing, whatever it is, has been either stunted or destroyed. One set of silkworms has been long living in an atmosphere of mild radium emanation and therein have passed through many generations. The colony's general departure from its original characteristics is curious, illustrating how local radio-activity may exert a real influence upon population. It is impossible to detail them all, but the altered sex distribution is striking. Originally the succeeding generations born contained males and females in about equal numbers. Now there are five males to every female, and there seems little doubt that the reason lies directly with the radio-activity to which the silkworms live exposed.

### THE LEPER.

THAT only 5 per cent. of the lepers in the British Empire are receiving any kind of care, and an even smaller number being treated by modern curative methods, are facts meant to stir our consciences as well as our hearts. None too soon, therefore, has the British Empire Leprosy Relief Association been formed with the object of making known the facts and endeavouring to obtain the means of extending the new methods of treatment to every sufferer. Nothing is more heartrending than the knowledge that deadly disease can be cured, yet that poverty or ignorance, or both, deny help to most of the sufferers. This is at present the case in regard to leprosy. Most lepers are poverty-stricken, and even before the time of Father Damien it was recognised that no one of the Corporal Works of Mercy made a more powerful appeal to human pity than the care of the leper. Something more than care, splendid as that is, is now possible, and recent research has opened the gates of hope to the unhappy creatures to whom, for so many centuries, hope has been denied. Leprosy is one of those intractable diseases which science in the end will assuredly learn to cure and even to prevent, but both money and co-ordination of effort are needed if that happy day is materially to be hastened.

### CARE OF PRE-TUBERCULOUS INFANTS IN PARIS.

IT is strange, but true, that the printing press, telegraph and other means for rapidly distributing information have in many cases failed to promote the adoption of new ideas in countries in which they did not originate. This is not merely a matter of national pride, although international prejudices certainly play a part. Perhaps some ideas are better suited to certain soils than others, but in so cosmopolitan a problem as that of tuberculosis, it is difficult to understand how a system which has worked well in one country should be almost unknown in others. The Grancher System consists of isolating infants as soon after birth as possible from their tuberculous parents, and bringing them up in healthy foster homes. It has been proved beyond doubt by Professor Bang of Denmark that when calves are taken from tuberculous cows directly after birth, the calves grow up as healthy as those born to non-tuberculous cows. The problem is, in short, one of infection and not of heredity, but it is easier to apply this teaching to cattle than to human beings. In an account recently given by Professor Bernard and Dr. Debré, it appears that the working of a modification of the Grancher System in Paris has already been very successful. Tuberculous women are informed of the dangers awaiting their unborn infants, and are persuaded to part with them at birth. Sometimes isolation of the infant begins only after it has been exposed to infection for several weeks, and in this connection a very important observation has been made. If an infant is removed from a tuberculous contact and does not die of tuberculosis within six months of such removal, the odds are that it will continue to live and thrive. Though the incubation period of fatal tuberculosis in adults may be a matter of decades, the incubation period of fatal tuberculosis in childhood is only a matter of weeks. The Grancher System is almost unknown in this country!

### AUDITING BODIES IN CHICAGO.

THE history of preventive medicine in Chicago is the life history of Dr. John Dill Robertson, one time Health Commissioner of Chicago. In his organ, *The Bulletin of the City of Chicago Municipal Tuberculosis Sanitarium*, he has some painfully plain words addressed to the general practitioner who is more concerned with the ethics of the medical profession than with maintaining the health, vigour and vitality of the community. This old-fashioned doctor is, according to Dr. Robertson, still to be found in America, obstructing and finding fault, whining over the competition of "osteopaths," "chiropractors" and "naprapaths," Dr. Robertson's advice to his colleagues is, in effect, to compete with other salesmen in the open market. The practitioner should sell the public the health idea—healthy practices and habits. There are only about 600 individuals to every doctor in Chicago, and Dr. Robertson advises each of his colleagues to take his 600 patients in hand in the following manner: "Go to your office to-morrow morning, take down your record and find how many people there are for whom you are the family physician. Take those that have been to your office during the past year; write them a letter; tell them you would like to have them drop into your office, that you would like to talk to them." The family doctor is then advised to make a physical audit of the ledger of life—to make, that is, a thorough medical examination not a "stick-out-your-tongue-let-me-feel-your-pulse" substitute for a medical examination. The interview is to conclude with an agreement between doctor and client—the potential patient—that the auditing of the life ledger is to be repeated at regular intervals. An annual contract like this with 600 clients—again let us avoid the term "patient"—will not only double and treble the doctor's income, but will give him the joy of knowing that he has saved lives and prevented sickness and pain.

### A TEN YEARS' TEST WANTED.

WE referred last month, in writing of Dr. Dreyer's discovery, to the need for caution and prolonged experiment before proclaiming victory over tuberculosis or any other disease which has successfully defied medical science in the past. There has since come to our notice a request from Dr. Crofton, Lecturer in Special Pathology, University College, Dublin, to which we gladly give publicity. For the past ten years Dr. Crofton has employed in prophylactic inoculation against tuberculosis an antigen which is a solution of the tubercle bacillus in benzoyl chloride—a preparation which contains, since it is a complete solution of the microbe, all the constituents of it. Dr. Crofton states that he has never known this solution to fail him in diagnosing a case of tuberculosis where the tubercle bacilli could not be found by other tests; and that he has not known a case of a member of a tubercular family developing active tuberculosis after being treated with this antigen. The request which Dr. Crofton makes is that his view, that by this method tuberculosis can practically be stamped out, should be put to the test in one area (town and country) for a period of ten years. No one will be likely to quarrel with his statement that a fair criterion of success in any method of treatment of a given disease is whether or no an effect can be produced in a given area on the Registrar-General's return of the deaths from that disease. It is a little surprising to learn, in view of the success which Dr. Crofton states has attended his own practice, that he has tried for many years, without success, to get some authority to adopt this method of prophylaxis.

### HOSPITAL WARDS AND PRIVATE PATIENTS.

THE need for better provision for those of the middle classes who find themselves unable to pay the present-day fees of the average nursing-home is well known. It is being met in a large number of institutions by admitting paying patients to the wards of the general and special hospitals. In this connection King's College Hospital has been criticised because, owing to lack of means, wards are closed to general patients, whilst some have been opened for such patients of the "middle classes" as are able to pay just sufficient to cover the cost of maintenance. To quote from a letter which has recently appeared in the Press written by the House Governor, following a similar communication from him in our June number, this has been done "as an expression of sympathy with the difficulties of this class in the time of serious illness." He adds that the opinion is expressed in many quarters that the combination should remain permanent in a large general hospital serving a great community where all classes are represented. At King's College the arrangements in these wards for paying patients are delightful, and ex-patients pay high tribute to the comfort, consideration and efficient nursing they have received. There is also a certain benefit to the nurses. The fact that there are very few in a ward, and that it is run somewhat on the lines of a nursing home, gives the nurses in the ward experience which, should private nursing afterwards appeal to them, will stand them in good stead.

### CONSOLIDATING HEALTH INSURANCE LAW.

LORD ONSLOW on behalf of the Ministry of Health introduced in the House of Lords on July 23rd a Bill to consolidate the enactments relating to National Health Insurance. The introduction of this Bill is in fulfilment of pledges given by Mr. Neville Chamberlain and his predecessor some months ago, and will be very warmly welcomed by all those concerned with Health Insurance administration. The procedure adopted in the case of Consolidation Bills is to send them to a Joint Committee of the two Houses, where they are carefully examined for the purpose of seeing that they do no more than consolidate the existing law without amending it. This procedure has been adopted in the case of the Insurance Consolidation Bill, and since this Bill has been urgently demanded for a long time past it may be anticipated that it will become law, if not before the House rises, at any rate in the autumn. We hope that the measure is the first of a series of Consolidation Bills dealing with the numerous groups of Statutes for the administration of which the Minister of Health is responsible.

### HEALTH AND NOISE.

A CORRESPONDENT of a daily contemporary has once more raised the question of the increasing noisiness of the world. He apparently finds the country as noisy in its way as the town, since he finds that "traction engines, heavy lorries, motor-cycles, and the loud aggressive type of horn are everywhere annoying to a degree both by day and by night." He would have the Ministry of Health "see to it." But what can the Ministry do? In the country the worst early morning noise is made by the cock, who congratulates himself upon having awakened to another day for his own delight and the ultimate benefit of the human race; but Whitehall has not yet seen its way to the suppression of cock-crowing by circular. There can, however, be no doubt that all large towns are becoming noisier—the late Lord Northcliffe declared that, in this respect, Brussels beats them all. It is hopeless to expect monastic calm in crowded streets—the mere volume of traffic, however quietly conducted, forbids. Londoners who do not often visit provincial towns are often astonished at the stream of traffic they find even in places of 50,000 inhabitants—they are under the impression that London is the noisiest and most congested place in England. That is far from being so, though even in London noise is increasing rapidly. Perpetual sound is not good for the nervous organisation—nor, for the matter of that, is the perpetual dodging of traffic.

